# Do food donation tax credits for farmers address food loss/waste and food insecurity? A case study from Ontario

**DOI:** 10.1007/s10460-019-09995-2

**Published:** 2019-11-05

**Authors:** Lesia Kinach, Kate Parizeau, Evan D. G. Fraser

**Affiliations:** Department of Geography, Environment & Geomatics, University of Guelph, 50 Stone Road East, Guelph, ON N1G 2W1 Canada

**Keywords:** Food waste, Food loss, Food insecurity, Food donations, Tax credits

## Abstract

To increase donations of nutritious food, Ontario introduced a tax credit for farmers who donate agricultural products to food banks in 2013. This research seeks to investigate the role of Ontario’s Food Donation Tax Credit for Farmers in addressing both food loss and waste (FLW) and food insecurity through a case study of fresh produce rescue in Windsor-Essex, Ontario. This research also documents the challenges associated with rescuing fresh produce from farms, as well as alternatives to donating. Interviews with food banks, producers and key informants revealed that perceptions of the tax credit, and the credit’s ability to address FLW and food insecurity, contrasted greatly with the initial perceptions of the policymakers who created the tax credit. In particular, the legislators did not anticipate the logistical challenges associated with incentivizing this type of donation, nor the limitations of a donation-based intervention to provide food insecure Ontarians with access to fresh, nutritious food.

## Introduction

Approximately one third of all food produced in the world for human consumption is lost or wasted (Gustavsson et al. 2011). In Canada, new research estimates that the value of avoidable food loss and waste from farm to fork per year is $49.5 billion; this equates to 3% of Canada’s GDP in 2016, or 51.8% of money that Canadians spent on food from retail stores that year (Gooch et al. 2019). It is estimated that the environmental impact of food waste in Canada is equivalent to 1.8 million hectares of wasted cropland per year and biodiversity loss equivalent to $26 million USD per year (Commission for Environmental Cooperation 2017a).

Notwithstanding these statistics, food waste is a difficult concept to define. The term ‘food waste’ is often used to describe food discarded at the retail or consumer stages, while ‘food loss’ typically describes food lost earlier in the food supply chain such as at farm or processing stages. In this paper, we use the umbrella term ‘food loss and waste’ (FLW) to refer to any wholesome edible material produced for human consumption that is discarded, lost, degraded or destroyed at any stage of the food supply chain (FAO 1981; see also KC et al. 2016). Although there is a moral tone to current discussions of the scale of wasted food, there are diverse individual, institutional, and economic causes of widespread food waste that render it a complex issue that cannot be easily solved (Mourad 2016).

The problem of FLW is often contrasted with the equally pressing problem of food insecurity. While the issue of hunger is often associated with developing countries, food insecurity also exists in wealthy nations like Canada. Household food insecurity is defined as “the inadequate or insecure access to adequate food due to financial constraints” (Tarasuk et al. 2016), and the most recent nationwide survey found that food insecurity was experienced to some extent by nearly 13% of Canadian households, or 4 million individuals in 2012 (Tarasuk et al. 2014a). The rescue and redistribution of surplus food is sometimes promoted as a way of addressing the coexistence of wasted food and hunger in affluent countries like Canada. Food rescue involves collecting safe, edible food that would otherwise not be eaten and redistributing it to charitable food organizations. The most common of these are food banks, which can be defined as “voluntary community organizations that solicit food and financial donations from the public and corporate sectors and distribute food assistance locally, according to whatever principles they have established” (Tarasuk et al. 2014b, p. 2). Food banks and other similar organizations often rely on donations from retailers, restaurants, or farmers to supply them with food that they then distribute via hunger alleviation programs.

In an effort to encourage more food donations, some jurisdictions have introduced tax incentives for donors. Several states in the US have implemented tax deductions or tax credits for donations to charitable food organizations (see Broad Leib and Rice 2017). Recently, these incentives have been targeted specifically to farmers to help increase donations of fresh, nutritious food and reduce FLW at the farm level. In Canada’s most populated province, Ontario, a tax credit for farmers who donated food to food banks was passed into law under the Liberal government as an amendment to the Local Food Act in November 2013. The Ontario Association of Food Banks (now called Feed Ontario) played a key role in supporting the development and passing of this legislation. Ontario’s Food Donation Tax Credit for Farmers provides farmers with a tax credit worth 25% of the fair market value of the agricultural products donated to eligible community food programs (OMAFRA 2019). Ontario was the first province to create such a tax credit, and soon afterwards Quebec, British Columbia, and Nova Scotia followed with similar policies.

One goal of these tax credits is to provide incentives for farmers to increase donations of healthy food, as concerns have been raised over the nutritional quality of food available at food banks (Jessri et al. 2014; Holben 2012; Irwin et al. 2007; Simmet et al. 2016). Equally, food banks have also supported this type of policy as a way to help farm producers reduce FLW (e.g. Food Banks Canada 2016). However, it is not clear whether or how such tax credits help to address food insecurity or FLW, and neither qualitative nor quantitative data measuring the impact of this policy is currently available. It is also important to consider factors that may inhibit the rescue and donation of fresh produce from farms despite the introduction of a tax credit, as well as alternatives to donations that may more effectively address the issues raised by legislators in devising this tax credit. This paper asks the following research questions: What is the role of Ontario’s Food Donation Tax Credit for Farmers in addressing FLW and food insecurity? What, if any, additional factors influence the effectiveness of the tax credit in increasing the amount of fresh produce donated from farms to emergency food services?

## Literature review

### Food loss and waste at the farm level

One of the main causes for loss/waste of fresh produce at the farm level in affluent countries is standards concerning the aesthetic characteristics of fruits and vegetables, such as their shape, size, colour, etc. (Bilska et al. 2016; Buzby and Hyman 2012; Hodges et al. 2011; Lucifero 2016; Mena et al. 2011; Neff et al. 2015; Priefer et al. 2016; Thyberg and Tonjes 2016; Willersinn et al. 2015). Anything that does not meet stringent visual/cosmetic standards is rejected by purchasers, despite being safe to eat (Lucifero 2016). Market fluctuations can also lead to farm-level FLW; entire crops may never be harvested if food prices are low and the cost of harvesting and transporting the produce is greater than the farmer’s expected return (Gunders 2012; Bloom 2010; Priefer et al. 2016).

Some strategies have emerged to reduce the loss or waste of fresh produce on farms. One initiative is the sale of off-grade ‘ugly’ fruits and vegetables through retail stores (Mourad 2016). Direct marketing, a strategy often used to distribute local food, could also help by acting as an alternative channel for the sale of food that fails to comply with grading standards (Priefer et al. 2016). However, some farmers worry that selling off-grade fruits and vegetables will drive down prices for the rest of their produce as well (Mourad 2016). Others argue that cosmetic standards should normally be relaxed as they are in years when the harvest consists almost entirely of imperfect produce (Aschemann-Witzel et al. 2015).

A common approach to reducing FLW is through food rescue, where imperfect or surplus produce is donated by producers to charitable organizations for redistribution to the food insecure. Significant volumes of food can be rescued from farms: a case study in the US found that in one season, 85,000 lbs (equivalent to 38,555 kg) of fresh produce that otherwise would not have been eaten were recovered and donated to emergency food organizations (Hoisington et al. 2001).

### Food insecurity, food rescue and food banks

Food rescue is often framed as an approach that will both reduce FLW and help address food insecurity. Food insecurity at the household level is characterized by inadequate access to food due to a lack of funds to purchase it (Tarasuk et al. 2016). Due to precarious employment, growing income inequality and reduced spending on social programs, the number of people who are food insecure and rely on food aid from the charitable sector is growing in affluent countries (Riches and Silvasti 2014). Food aid is commonly distributed through food banks, some of which act as a central hub and redistribute donated food to other organizations that distribute the food aid directly to those in need. In North America, food banks began to emerge in the 1980s as a temporary measure to supply emergency food relief but have become the key source of charitable food distribution (Kicinski 2012; Starkey et al. 1998).

As food banks largely rely on food donations and food rescue programs, they face a number of challenges with supply. Food banks must constantly grapple with the unpredictable nature of food donations that can result in over or under supply from one day to the next (Reynolds et al. 2015). Limited storage capacity is also a barrier (Campbell et al. 2013). The unpredictability of food donations can result in situations where food banks either fall short on meeting the demand for food assistance (Oderkirk 1992; Wilson and Steinman 2000) or are overwhelmed with food donations and unable to distribute them all (Campbell et al. 2013).

Relying on food donations has also led to concerns surrounding the nutritional quality of food that is given to food bank users (Tarasuk and Eakin 2003; Rock 2006; Irwin et al. 2007; Mourad 2016). In their review of the literature on the nutritional quality of food distributed by food banks, Simmet et al. (2016) found that most of the organizations studied could not provide an adequate amount or variety of food for a well-balanced diet (see also Bazerghi et al. 2016). Fresh produce is of particular concern: one study in British Columbia found that food bank users had low produce intake and concluded that it is important for food banks to find ways to make fruits and vegetables available to users (Holben 2012).

In addition to donations from food retailers, food banks may engage with food rescue initiatives that recover fresh fruits and vegetables from farms to increase their supplies of fresh produce. Sometimes this occurs through gleaning, which involves a group of volunteers harvesting surplus or unmarketable produce and delivering it to food organizations for distribution (Hoisington et al. 2001; Lee et al. 2017). In other cases, farmers donate crops they have harvested and packaged themselves. While providing healthier foods is a goal of many food banks, they may face additional challenges in handling, storing and distributing fresh produce because of its perishability (Campbell et al. 2013).

### The complexity of connecting FLW with food insecurity

While many organizations attempt to address FLW and food insecurity together through food rescue, in reality, the relationship between these two issues is complex. On the one hand, donating surplus food to food banks is widely accepted as a means to reduce FLW while alleviating hunger (Thyberg and Tonjes 2016; Lee et al. 2017; Stuart 2009; Bilska et al. 2016; Vlaholias et al. 2015). This approach is highlighted in the USEPA’s food recovery hierarchy that prioritizes rescuing food as a FLW strategy second only to reducing surplus in the first place. However, the appropriateness of trying to solve these two problems at the same time is contested by scholars arguing that food rescue and redistribution through food banks is not a long-term solution to hunger, and that governments need to take action to address the root causes of the issue (Riches and Silvasti 2014; Tarasuk et al. 2014b; Riches 2011; Tarasuk and Eakin 2005).

There can also be a stigma around receiving emergency food aid that has been donated as surplus or ‘waste’ food (Caraher and Furey 2017). In the face of short supply, food banks cannot discriminate when accepting donations and thus in many cases, “food assistance can become defined as that which the corporate sector cannot retail” (Tarasuk and Eakin 2005, p. 183). In other words, food bank recipients may be receiving what otherwise might have ended up in the garbage. When Canadian food insecurity policy entrepreneurs were asked to comment on a hypothetical proposal for a tax incentive for retailers who donate surplus food to food banks, several of them renounced the proposal on the basis of human dignity. They called upon the human right to food to explain their position (McIntyre et al. 2016a). Despite the good intentions of food banks, some scholars argue that handing out surplus or ‘wasted’ food to hungry people does not promote the dignity that is required to fulfil the human right to food.

While there is critical discussion of the problems with retail donations to food banks (Fisher 2017; Tarasuk and Eakin 2005), and more specifically, the idea of creating a tax incentive for retail food donations (McIntyre et al. 2016a), there is little academic discussion of farmer donations in this debate. One of the key criticisms of the emergency food system is the poor nutritional quality of the food it distributes, given its reliance on donations (Tarasuk and Eakin 2003; Rock 2006; Mourad 2016), and levels of fresh produce intake amongst food bank users are of particular concern (Holben 2012). Receiving donations of fresh produce from farmers, then, might lead to an improvement in access to nutritional foods. However, barriers to donation may prevent this intervention from improving the nutritional quality of food bank offerings. Given that Ontario’s Food Donation Tax Credit for Farmers has prompted other provinces to follow suit, it is important to understand how the intended outcomes of this policy compare to the actual outcomes as experienced by producers and food banks.

## Methods

**Fig. 1:**
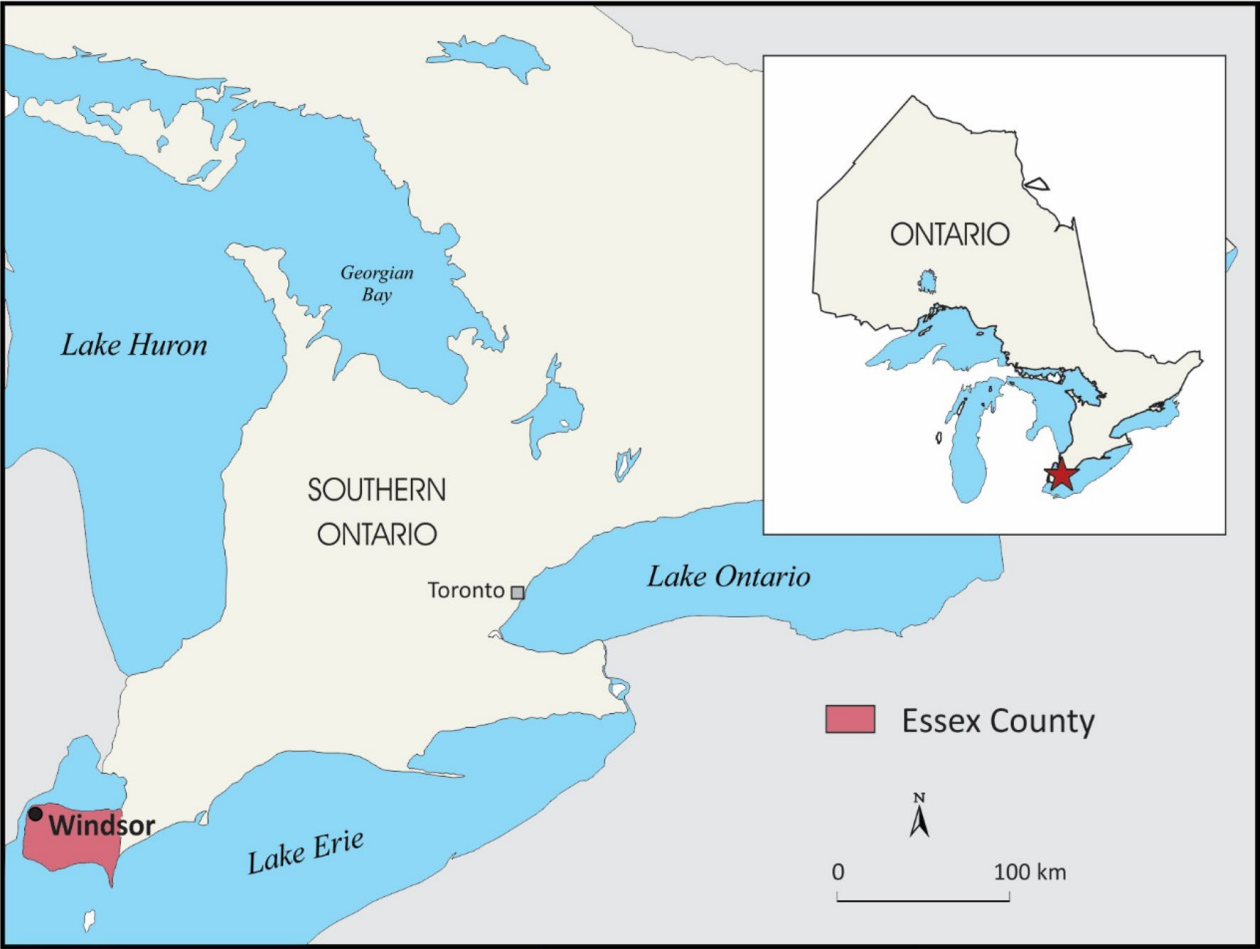
Map of Ontario showing the location of the city of Windsor and Essex County

This research uses a case study approach (Bryman et al. 2012; Neuman 2014) and is focused on donations of fresh produce in Windsor-Essex, Ontario. Windsor and Essex County is located in Southwestern Ontario (Fig. 1), home to 69% of Southern Ontario’s field vegetable production and 84% of greenhouse vegetable production (Kubursi et al. 2015). Windsor-Essex also has a well-established food rescue network. Two programs run by large non-government organizations collect fresh produce from farms and greenhouses and distribute it to food banks and other organizations in the area. The central hub of the Windsor Essex Food Bank Association also distributes collected food to other member food banks. Since the implementation of Ontario’s Food Donation Tax Credit for Farmers in 2014, some of the organizations involved in food rescue have been issuing tax receipts to the producers who donate food. Windsor-Essex is therefore an ideal place to examine the perceptions of both farmers and food bank operators of the tax credit and the challenges surrounding donations of fresh produce.

In order to understand policymaker perceptions of the tax credit, the lead author for this paper searched and extracted transcripts from the Ontario Hansard, which is an official, near-verbatim transcript of the debates recorded during parliamentary sessions. Using the Advanced Hansard Search (http://hansardindex.ontla.on.ca/), 37 transcripts were found that included debates regarding the Food Donation Tax Credit for Farmers between late 2007 (39:1) and early 2018 (41:2). The lead author also conducted semi-structured interviews with 11 food bank representatives (paid/volunteer staff), 11 producers/field growers, and 10 key informants (including six academics and four organizations) in summer 2017. The organizations interviewed were devoted to promoting food security in Canada and focused on advocacy and education rather than front-line service like food banks. Research participants were contacted through online directories and snowball sampling. Both Hansard transcripts and interview transcripts were analyzed with NVivo qualitative analysis software using a combination of open and axial coding methods, drawing on grounded theory analytical traditions.

### Limitations

Even though this study was based in Ontario, where the farmer tax credit had been in place the longest of any jurisdiction in Canada, three tax years is a relatively short period and several farmers were unfamiliar with the policy. The timing of data collection in late summer was also a limitation, as this is a very busy time for produce growers and so some prospective research participants were unavailable. No representatives from greenhouse growing operations responded to requests for interviews, despite repeated attempts at contact. Several of the food bank representatives said they received donations from the greenhouses in the area, and so the exclusion of this producer sector represents a limitation to this study.

## Results and discussion

### Perceptions of the tax credit

Policymakers’ perceptions of the food donation tax credit, both before and after the legislation, were overwhelmingly positive. One of the main benefits they highlighted was that the tax credit would support farmers financially and provide an incentive to donate food rather than plow crops under. As Robert Bailey, the Member of Provincial Parliament (MPP) from the Progressive Conservative Official Opposition who introduced the idea of a tax credit for farmers, proclaimed:

My proposed legislation will provide a financial incentive for producers to donate, that will provide producers, at minimum, with a tax credit which will help to offset some of the costs associated with growing and harvesting fresh produce, while in many cases it will provide producers with a net financial benefit for the donation of those surplus food products. This proposed tax credit would reduce the producers’ tax burden, which in turn should provide a strong incentive to make that donation. (MPP Robert Bailey, 16 September 2010)

The claims made in the above excerpt were echoed by several MPPs of all political stripes. While policymakers lauded the tax credit as a great incentive, most producers thought that the tax credit value was too low and needed to be increased in order to have a meaningful impact. One producer explained:

Well I think it’s a good idea, but I don’t think it should be at 25%, I think it should be at 100%… giving a 25% tax credit was almost insulting. Like it kind of tells me that my work is not valuable. Or it’s only worth a quarter of anybody else’s work. (Producer 1)

Others accepted that 25% was “better than nothing,” but echoed the need to increase the tax credit in order to better offset the costs associated with donating:

At least you get a tax write-off, otherwise it would be 100% loss, right? […] It’s better than nothing, that’s all it is. It should be a better—‘cause you don’t even get back your cost, you know what I mean. It would be nice to break even in our cost, which should be half—50%—but I don’t make the rules. (Producer 8)

Evidently, there is a disconnect between the policymakers’ perceived outcomes of the tax credit and the reality on the ground for producers, which has implications for the ability of this type of policy to actually support farmers.

There were also discrepancies between policymakers’ and interviewees’ perceptions of the benefit of the tax credit for food banks. In particular, debates in the Provincial Legislature reveal that the main impetus for this policy was to increase food banks’ supply of nutritious food at a time when donations from the manufacturing sector were falling. One MPP from the Official Opposition highlighted the motivation for the tax credit and the expected outcomes:

The reason why I hope we can pass this bill… is because of the urgent need that we see today…What we need today is not more review and not more study, because I will refer to the fact that similar programs have been set up elsewhere. What we need is action. There is a desperate need for food.

[…]

Who’s going to benefit? I’ll just summarize by saying what will happen if we have this tax credit… (1) We could increase the supply of nutritious food to low-income families in Ontario; (2) we could reduce the level of agricultural surplus from Ontario farms; and (3) we could support local agriculture by reducing the losses for primary producers. (MPP Elizabeth Witmer, 16 September 2010)

Another MPP from the governing Liberal party predicted the measure would increase donations to food banks, while also supporting producers:

By passing this [bill], were this to come to pass, it’s going to provide a much broader base for potential donations to the food bank system that we have here in Ontario. It’s going to encourage more people to donate, and it’s going to encourage a broader base of donations in the first place. I’m supportive of any concept that’s going to help our food banks, any concept that also is going to help our agricultural industry. (MPP Kevin Daniel Flynn, 16 September 2010)

In contrast, most producers interviewed indicated they would donate or currently donated without using the tax credit, and that it did not have a significant influence on the frequency or volume of their donations to food banks. As one producer noted:

No… It doesn’t influence—and that’s why I said earlier, I’m not growing a crop or planting acres, or planting a crop that in the back of my mind a certain percentage of that crop will be donated for using this program. That’s just… no. That doesn’t come into the picture at all. (Producer 10)

Only one producer mentioned that his donations would be minimal without the benefit of the tax credit. This is likely because farmers were already donating before the tax credit was introduced and generally were motivated out of a desire to both support their community and to see their produce distributed for consumption rather than plowed under. These sentiments resonate with social science theory called the “moral economy of food” that was articulated by Scott in the now-classic paper “The Moral Economy of the Peasant” and Thompson’s equally seminal “The Moral Economic of the English Crowd” (Scott 1977; Thompson 1971). These publications posit that for many people, food does not belong in the normal “market-driven” economy where production and distribution are necessarily caused by competition between firms seeking to maximize profit. Rather, the idea of the moral economy acknowledges that many people treat food as a special kind of good to which everyone has an unalienable right. The literature on the moral economy of food posits that moral, ethical or even spiritual conflicts arise when some groups start to treat food as a market good and then establish trading agreements to distribute food based on financial incentives. In particular, shifting into a market-based system can undermine the actions of people who previously assumed that food belongs to the moral economy. Hence, most of the literature on the moral economy of food shows that conflicts can arise out of moral outrage over a transition to a market-based system and this moral conflict may be far more consequential in the participant’s eyes than any considerations associated with the materiality of food itself (Fraser and Rimas 2011; Fraser and Rimas 2010). This discussion relates to the tax credit in that prior to the credit’s establishment, farmers who donated their food did so out of a sense of moral obligation with no expectation of a market-based reward. When the incentive arrived, and when farmers realized how little it was worth financially, the tax credit may have had the effect of simply devaluing their previous actions.

Food bank representatives generally viewed the tax credit positively and believed that it provided a financial incentive to farmers to help them donate rather than plow under their crops. However, one representative acknowledged that the tax credit has had limited financial benefit and had probably not influenced the quantity of donations that they receive:

And I don’t know if it has incentivized [producers’] donation—they were donating to us anyway. I think it’s a bonus, and I think it’s good for them, but I don’t think it would have stopped them from donating. Cause I think they feel good that they’re not dumping that product… If they’re harvesting it anyway, and that’s the thing. If they have to harvest that product anyway and they’re grading it out—but if they have product on the field and they knew they were gonna grade most of that product out, they would just plow it under. Cause it would probably cost them much more to harvest it than they would get on their tax rebate. (Food Bank 6)

There is clearly a discrepancy between policymaker perceptions of the benefits to food banks and respondents’ descriptions of the actual outcomes, as their responses suggest that this policy has not significantly changed producers’ donation practices. There was very little discussion in the House on the limitations of the tax credit, which indicates that policymakers were mostly focused on the potentially positive aspects of this policy. Less consideration was given to the logistical challenges associated with increasing farm donations that could prevent the use of the tax credit, or the implications of a tax credit on the capacity of food banks to accept and distribute increased volumes of perishable donations.

### Logistical challenges with farmer donations of fresh produce

The main challenges that respondents described in terms of donating farm produce included the costs of donating, arranging the transportation of donations, and food bank capacity to accept perishable donations. The producers’ costs associated with donating fresh produce included the costs of packaging materials and the labour involved with harvesting and sorting produce that would be given away for free:

…but the thing that will be a challenge, and it’s always a challenge, is the cost of donating. Like they still go out in flats, they still go out in boxes, you still have your labour, picking them, sorting them, and all that… and as our labour prices are going up, 28% this year and another 8% the year after that, like it puts pressure on all sides, right. (Producer 7)

One producer noted that the costs associated with readying food for donation were borne disproportionately by their sector:

I guess, if we’re going to donate produce to a food bank, why should it always be on the so-called backs of the primary producer? Why should we be taking an economic hit just so we can feed some people? (Producer 10)

While producers identified donation cost as a challenge, most of them indicated that the tax credit was not influential in their decision to donate and that they would still donate without it.

Another important barrier to several producers was transportation. In many cases, the ability of food banks to arrange pick-up at the farm gate with their own truck was the determining factor for donating. These producers cited inconvenience, fuel cost, and lost productivity (being away from the farm) as reasons why they would not transport donated food themselves. Food bank representatives also noted their constraints in coordinating transportation for the collection of fresh produce from dispersed farms and transporting it to organizations that can distribute it. One food bank representative explained:

So, if we could have a system in place, particularly when it comes to food waste, where we could have vehicles where we could access to move around southwest Ontario, I think there is a great opportunity to have a food swapping network in southwestern Ontario. […] I think we could probably rescue even more food if we have places for it to go to, and the systems in place to get it there. Transportation is the biggest challenge. (Food Bank 6)

Innovative models are being developed to facilitate the transportation of surplus food to those in need: in May 2018 Second Harvest, the largest food rescue organization in Canada, launched an online network called FoodRescue.ca to connect food businesses with “rescuers” from charities, not-for-profit organizations and schools. However, whether this type of platform assists farmers in donating fresh produce is not yet clear.

Both producers and food banks noted that the capacity of food banks to accept and handle perishable donations posed a barrier. As one producer noted, this limitation meant that produce available for donation can sometimes go to waste:

And that’s the issue, is it’s perishable stuff, and I’m realizing that food banks don’t have big coolers, they don’t have the refrigeration capacity […] so you don’t want to overbear them because I can’t say oh here, I’ve got 30 bins… like what are they gonna do with 30—they can only handle 4 or 5 every day or two… and if I wait a week, well then I might as well dump them because they’re mush. (Producer 10)

Food bank representatives also described how the unpredictable nature of the supply, quality, and quantity of fruit and vegetable donations from producers posed challenges. All but one producer acknowledged that donating is an afterthought for any produce that is still edible but cannot be sold. Several producers expressed that they would rather give their surplus or ‘seconds’ to people than dump it back into the field or plow it under. For food banks, this poses a challenge because they cannot expect to receive a steady and predictable supply of fresh produce. In addition, the variety of produce donated also depends on the producer, which means that food banks sometimes receive mostly one type of fruit or vegetable at a time. The tax credit does not effectively address this issue of donation supply.

When food banks do receive donations of fruits and vegetables from producers, the quality of those donations can create significant challenges for them. Most food bank representatives said they do not refuse any donations; they sort through them and sometimes must dispose of substantial amounts because it was rotten or otherwise unfit to distribute. However, accepting poor quality donations has implications for these charitable organizations, particularly related to the cost of disposal. As one food bank highlighted:

But there are some times where we get a little bit dumped on, where some greenhouses have product sitting around that really isn’t very nice. Usually we get number 2s, sometimes we get much better than that. Sometimes we get certain groups that will send us stuff that’s almost rotten. So at that point, we’re faced with dumping charges, and we try to limit that. (Food Bank 9)

One food bank representative did mention that they have refused donations in the past, but most did not want to turn any away because they do receive some good quality produce mixed in with things that they then must dispose of. In particular, many food banks do not reject undesirable (inedible, unhealthy) donations as a matter of policy for fear of losing access to future donations from donors whom they rely upon for their supply (Tarasuk and Eakin 2005; Commission for Environmental Cooperation 2017b; Campbell et al. 2013).

Similarly, the quantity of donations from producers also poses similar challenges to food banks. They sometimes received such large quantities of produce that they could not distribute all of it—either to food bank users or other agencies—before it spoiled. It is, therefore, important to consider how large-scale donations of produce might offload food waste disposal from producers or corporate donors to food banks, especially when the donated food is highly perishable. The Ontario Food Donation Tax Credit for Farmers was designed to encourage more food donations, but it does not appear to address the existing challenges food banks face with accepting and distributing large quantities of perishable food items. Accepting any and all donations still shifts the cost of disposal from donors (producers, corporations) to food banks or other charitable organizations (Mourad 2016; Suschnigg 2012).

Similarly, the administrative logistics associated with participating in the tax credit were not borne primarily by producers. Food banks had to determine the fair market value of the food being donated, which posed a challenge to them. However, only a few of the interviewed food banks issued receipts, in part because of the administrative burden this task represented, and in part because many received farm donations through a distributing organization and not directly from producers.

Some food banks are devising ways to handle large volumes of fresh produce through innovative models of direct delivery to users (e.g. Campbell et al. 2013). The Farm to Food program launched in 2018 by Feed Ontario both rescues food and builds skills: for example, graduates of the culinary skills course at the Unemployed Help Centre in Windsor use donated produce to create healthy meals that are frozen and distributed to hunger-relief agencies across the province. Feed Ontario is also working to address this issue by providing food banks with funding through their Capacity Building Program. Research has been done to determine the optimal schedule for maximizing fresh produce rescued by gleaners (Lee et al. 2017), but further research could explore optimization and best practices for fresh produce rescue and distribution without the help of volunteer gleaners.

### Debates about food rescue as a solution for food insecurity

There were discrepancies between the perceptions of policymakers, food bank representatives, and key informants with respect to the impact of this tax credit on food insecurity in Ontario. Most policymakers believed that by increasing donations of nutritious food to food banks, this tax credit would in turn help people in need gain access to fresh, healthy food. The then-current Minister of Agriculture, Food and Rural Affairs noted:

We know there are folks in all of our communities across the province of Ontario who face some challenges, and we want to make sure they have the opportunity to have access to fresh, nutritious food, and the food donation tax credit that was championed by Mr. Bailey is making that happen. (Hon. Jeff Leal, 2 April 2015)

Similarly, another MPP from the relatively left-wing New Democratic Party noted:

There are literally hundreds of thousands of people here who rely on food banks. If we can give them even some fresh fruit and vegetables that would otherwise be wasted, it is an absolutely important thing to do. (MPP Michael Prue, 23 April 2013)

Most food bank representatives also suggested that FLW and food insecurity are interrelated problems that could be addressed together by using excess produce to feed hungry people. One explained:

Well they are connected, cause when we can take the food waste of one and put it on the plates of the people who have food insecurity…we can connect that waste, what we would call waste from the—I hate the word waste—that food, that still edible product that can’t be sold, we can connect that through an agency like ours to the people who need it. (Food Bank 2)

While food bank representatives generally thought connecting FLW and food insecurity would produce positive outcomes, some also understood that connecting these two issues could be problematic, particularly if it led to the distribution of lower-quality foods to low-income people. One food bank representative explained:

If I look at something and I wouldn’t eat it, a tomato that’s mushy, the same with any other vegetable or fruit, then I’m not gonna give it—I’m not gonna say whoa, you poor, you take it or shut up. No, that’s not our philosophy, and I don’t think it should be our philosophy as a government or as a community. (Food Bank 10)

Echoing this view, the majority of key informants (academics and food security organization representatives) expressed negative views toward the tax credit and maintained that it is an ineffective policy for addressing food insecurity. Specifically, key informants pointed out that many people experiencing food insecurity do not use food banks, and those who do are given such limited quantities of food that it does not impact their overall food insecurity or nutritional health. They expressed that the tax credit does not address the root causes of food insecurity, which are low income and insufficient income supports. As one key informant noted:

Adding a bag of carrots to the bin in [food banks] won’t change their impact on household food insecurity. I mean, the reason they don’t have a bigger impact is because they’re not actually addressing the problem. And so, you know tinkering with incentives to increase donations is just, I think it’s just a complete waste of taxpayers’ money. (Key Informant 3)

Key informants also cautioned against conflating FLW and food insecurity and thought it inappropriate to simply give surplus or ‘waste’ food to low-income people. As one respondent remarked:

…what is the moral basis for using surplus or wasted food to feed hungry people? What does it feel like to be on the receiving end of charity? I mean, what are people’s rights, you know given that we’re all rights holders and poor people and low-income people in particular, don’t they have the same right as you or I to go into a store like anybody else and purchase the food of their choice? (Key Informant 2)

This commentary highlights concerns about the morality and dignity of food donation as a systemic means to address food insecurity and hearkens back to the discussion above about how conflicts arise when food exchanges, which are often thought of as being driven by a “moral economy,” are treated with financial incentives.

However, not all key informants dismissed the tax credit outright. Even if they did not agree with the principle of the tax credit as a food security solution, they acknowledged that it did have the potential to make healthier food available to those who do use food banks and support farmers in some way. This ambiguity is exemplified by the following response:

Well, I suppose I think lots of different things about it. I suppose if it’s food that farmers wouldn’t be able to sell otherwise, then it’s a good thing for them to get a tax credit to donate it. Certainly, people who use food banks have really low intakes of fresh fruit and vegetables and meat, and so they need lots of help in accessing good food, good quality food… The problem is… I think symbolically, when the government is saying you know food banks are here to stay so we’re gonna give farmers credits for their unused food, or the food that they can’t market then… it’s another band-aid, and by government actually saying we’re gonna support this, you know on the surface it looks like a good thing but, it feels like a further layer of institutionalization of food banks, which are already deeply entrenched. (Key Informant 4)

It is important to note that some policymakers also expressed ambiguity in their perceptions of what the tax credit could achieve. MPP Robert Bailey, a member of relatively right-wing Conservative party that at the time was the Official Opposition, introduced the tax credit and acknowledged on a few occasions that this initiative would not solve hunger completely. Another MPP from the New Democratic Party highlighted that although the tax credit is a good initiative, it does not address the root causes of food insecurity:

The tax credit for farmers is good. It’s something that I support. But quite clearly, and I think all members in this House should recognize, this is actually just a band-aid solution, because people’s reliance on food banks and food charity remains the elephant in the room. This government’s failure to address food security for people living on low incomes in Ontario remains deplorable. The real problem remains that too many people in this province simply cannot afford any kind of food at all, let alone nutritious food, let alone local food or sustainably-grown food. Too many people just simply cannot afford food for themselves or their families. (MPP Jonah Schein, 4 November 2013)

This ambiguity in policymaker and key informant perceptions of the tax credit highlights the complexity of positioning food rescue as a key solution to food insecurity. Namely, it shows how an initiative to support farmers and increase fresh produce offerings at food banks—both positive goals—is not simply a ‘win–win’ because it does not address the issues behind why food banks are needed in the first place. This divergence of views on the relationship between FLW and food insecurity is reflected in the literature. Some authors support rescuing food and redistributing it to the hungry ‘in the meantime’ until there are major changes to social policies (Stuart 2009; Bilska et al. 2016; Vlaholias et al. 2015), while others stand firmly against conflating the two issues and trying to solve hunger with ‘waste’ food (Riches and Silvasti 2014; Tarasuk et al. 2014c; Riches 2011; Tarasuk and Eakin 2005). Divergent views on connecting FLW and food insecurity have also been observed among food security policy actors. For example, in their study of perspectives on creating a tax incentive for grocery stores that donate to food banks, McIntyre et al. (2016a) found that some participants rejected this proposal outright, while others supported it. These contrasting perceptions demonstrate the complexity of the issues of food waste and food security, as well as the complexity of finding solutions that do not treat low-income people as an outlet for ‘waste’ food.

The final point that several key informants took issue with was the lack of evidence that shows the tax credit was actually increasing food donations and helping address food insecurity. One key informant highlighted:

So, these legislations are not just neutral, they are negative. They entrench food banks, they get people to feel like the government’s done something—oh look, look what the Ontario government did. It instituted this great new legislation, which potentially didn’t even increase donations because nobody has any proof that it has. So it’s a problem, that’s the wrong problem… the wrong solution to the wrong issue. (Key Informant 5)

While there is seemingly no evidence that shows the tax credit would be effective in achieving the outcomes MPPs hoped for, some key informants noted that there is evidence in the literature that suggests income-related social policies can help reduce food insecurity (as discussed below). Key informants believed that for the government to champion this tax credit as a way to address hunger, more evidence is needed to show its effectiveness in helping food banks as well as the food insecure.

### Alternative interventions for addressing FLW and food insecurity

Given the limitations apparent in the tax credit system, respondents were asked to identify what types of approaches (other than donating to food banks) could help address FLW and food insecurity. Several different types of actions were proposed to address both problems, including actions that involved producers selling—rather than donating—fresh produce, and actions that involved government interventions or systems change.

Currently, most initiatives to reduce farm-level FLW (and simultaneously address food insecurity) involve an approach where producers donate food to food banks (Bilska et al. 2016; Dou et al. 2016; Garcia–Garcia et al. 2015; MacRae et al. 2016; Papargyropoulou et al. 2014; Schneider 2013), but this model often fails to support producers. In contrast, the most common approach to reducing FLW mentioned by respondents was to create opportunities for farmers to sell produce that is surplus or second-grade. More specifically, key informants suggested that producers could sell surplus or seconds to food banks or other organizations that would purchase it. One key informant explained:

…I think what is better is for food banks to buy fresh fruit and vegetables from farmers that they can’t sell, potentially at a reduced rate. Like what I’m proposing—and what I proposed to [the Ontario Ministry of Agriculture, Food and Rural Affairs] but they didn’t implement was… Farmers could sell directly to community food programs and they could get a tax break for that… I think food banks could, instead of asking you to donate a can, could say give us the money that we need to buy healthy food. There’s a way of changing the paradigm… (Key Informant 5)

Indeed, some producers expressed interest in selling their second-grade produce if there was a market for it, and even developing new products that would use second-grade produce, rather than giving it away for free. As one producer explained:

…we never had an outlet for second peaches to make juice or something… if you could develop a product that used that, like if at the end of the day we had like a really nice system where it would just go and it becomes something else, that would be wonderful. But somebody’s gotta develop all that and an easy way to do it. Because at the end of the day, we’re just kind of maxed out on what we can do and just barely make it. (Producer 1)

The key challenge noted here is the time and energy that may be necessary for a producer to find another market to sell surplus or second-grade produce.

Creating opportunities for producers to sell surplus or second-grade produce was also a key action proposed by respondents to help address food insecurity. More specifically, several key informants, including one food bank representative, highlighted the need to move away from the conventional food bank model to a model that would involve purchasing fresh produce from farmers at a fair price, enabling people to purchase low-priced or sliding-scale-priced food as customers rather than be treated as social service clients. These participants preferred to see a community-centred approach rather than one focused solely on low-income individuals. In Ontario, organizations like The SEED and FoodShare have built relationships with producers to purchase fresh produce directly for their programs. Of course, such arrangements require that food banks and other non-profits have the resources to purchase food from farmers, which is often a barrier. This is the case, for example, with the Farm to Family program in California, which makes fresh produce available to food banks at a greatly reduced price. However, even at discounted prices, few food banks have been able to afford this produce (Gunders 2012).

To address the problem of food insecurity, the most common respondent suggestion was to increase income-based government support by increasing social assistance rates, implementing a guaranteed basic income, and/or implementing more income-based social policies. This was proposed by nearly all key informants and a few food bank representatives, who identified income as the main determinant of food insecurity. As one key informant explained:

We need social programs that enable people to have enough money to meet basic needs and among the basic needs is food. So, we know for example in Ontario that about two-thirds of households reliant on social assistance are food insecure. Why? Because social assistance benefit levels are too low for most people to make ends meet. So, there’s a perfect example of something that falls under the provincial government’s purview, and is effectively legislated food insecurity. What should they be doing, rather than issuing tax credits, you know ostensibly for some kind of a diversion of food waste to producers and contemplating expanding that to the food manufacturing or retailing industry, like they need to be improving the incomes of people at the very bottom of the economic spectrum in this province so that they can manage. (Key Informant 3)

These views correspond with evidence in the literature that income-related social policies have the potential to reduce food insecurity rates. For example, Ionescu-Ittu et al. (2015) found that income supplementation through the federal Universal Child Care Benefit ($100 monthly for each child under 6 years old) significantly reduced food insecurity for beneficiary households and had a greater impact for vulnerable groups (low-income or single parents). Other research suggests that a guaranteed annual income is an effective strategy for reducing poverty, especially for low-income earners who are food insecure (McIntyre et al. 2016b). There is also evidence that suggests food insecurity rates among social assistance recipients are heavily impacted by the strength of such provincial policies (Loopstra et al. 2015). As for other suggestions, half of the key informants suggested that governments need to adopt more joined-up policies that address interrelated social issues such as housing, food, and health in a more holistic way.

Raising the minimum wage was also advocated by a few food bank representatives and half of the key informants, who highlighted that many food insecure households actually depend on wages. Interestingly, this conflicted with producers’ responses, who spoke of the challenges increasing wages would pose to operating their farms.

To address the problem of FLW, some key informants advocated for a systems approach that would include multiple tools and strategies across all levels of the food system, including some interventions under the purview of various levels of government. Examples included initiatives such as changes to date labels, banning organic waste in landfills, and composting at scale. Taking a more holistic approach to addressing FLW was also seen as a way to make the overall food system more ecological. As one key informant noted:

When we talk about food waste, we need to think about the food system at every juncture. Reducing waste is about finding economic efficiencies but also, from a system perspective, about ecologically-resilient food production and consumption. We need to move in an ecological direction. (Key Informant 6)

Addressing an issue as complex as FLW requires an approach that includes high-level changes, whether through government interventions or other broad-scale changes in the food system.

## Conclusions

The first research question motivating this analysis was the following: What is the role of Ontario’s Food Donation Tax Credit for Farmers in addressing FLW and food insecurity? This research reveals important contradictions in stakeholder perceptions of Ontario’s Food Donation Tax Credit for Farmers and its role in addressing FLW and food insecurity. Most notably, the views of policymakers and research participants on the prospective and actual outcomes of this policy contrasted greatly. Policymakers spoke highly of the prospects of this tax credit to benefit farmers, food banks, and food insecure Ontarians, believing that this policy could address food insecurity and FLW simultaneously. In contrast, this research finds that the tax credit was not a significant factor in producers’ decision to donate, and most producers thought the credit should be worth more than 25% of the value of the food in order to offset the costs of donating. Furthermore, key informants saw issues with the way that the tax credit conflated FLW and food insecurity, and how it was presumed to effectively address these problems when there is no evidence that it does so. With other jurisdictions in Canada adopting a similar tax credit, there is a need for more research to assess the effectiveness of such policies in reducing FLW and in supporting farmers, food banks, and the food insecure. More specifically, there is a need for studies to investigate changes in the quantity of food donations as a result of this type of farmer tax credit.

The second research question was as follows: What, if any, additional factors influence the effectiveness of the tax credit in increasing the amount of fresh produce donated from farms to emergency food services? Our research illuminates the key challenges and barriers surrounding the rescue of fresh produce from farms. Respondents noted that such challenges were not effectively addressed by the tax credit. For both producers and food bank representatives, the perishability of fresh produce was at the root of most of the challenges. In particular, food bank capacity for storage and distribution was a common challenge identified by respondents. For producers, this limited how much produce they could donate at a time, regardless of how much they had available for donation, and for food banks it limited how much fresh produce they could accept without being overburdened. While food banks welcomed much needed donations of fresh fruits and vegetables, the high perishability of these foods led to food banks absorbing the disposal costs of spoiled, inedible donations. This phenomenon challenges the assumption that donating to food banks inherently reduces FLW. In order to address perishability concerns, food banks receiving increased amounts of donated produce will need to be provided with enhanced food storage infrastructure, transportation support for donations, and logistical assistance.

Finally, this research highlights the need to find solutions beyond donation to reduce the loss of fresh produce on farms and address food insecurity. While several alternative actions were proposed by respondents, creating opportunities for producers to sell fresh produce instead of donate it was supported by producers, food bank representatives, and key informants as an intervention with the potential to address both FLW and food insecurity. This would require modifying the donation-dependent model to a system where producers are compensated for their produce, regardless of whether it is second-grade or top quality, and making this food available to the broader community rather than only to low-income individuals. However, it is important to ensure that any initiatives that involve selling second-grade produce would not have unintended consequences for producers, such as driving down prices for their number one grade. More research is needed to examine the viability of secondary markets for surplus or second-grade produce, and to investigate other innovative ways to support producers, reduce FLW on farms (e.g. Johnston 2018), and alleviate food insecurity at the same time.

## Abbreviations

FAO: Food and Agriculture Organization of the United Nations
FLW: Food loss and waste
GDP: Gross domestic product
MPP: Member of Provincial Parliament
OMAFRA: Ontario Ministry of Agriculture, Food and Rural Affairs
USD: United States dollars
USEPA: United States Environmental Protection Agency
